# Identification and Multifunctional Properties of Lactic Acid Bacteria Isolated From Fermented Turnip Juice

**DOI:** 10.1002/fsn3.70272

**Published:** 2025-05-20

**Authors:** Ilkin Yucel Sengun, Husniye Tansel Yalcin, Berna Ozturk, Aysegul Kirmizigul Peker, Gulden Kilic, Yigit Terzi, Yunus Yahsi

**Affiliations:** ^1^ Engineering Faculty, Food Engineering Department Ege University Izmir Türkiye; ^2^ Science Faculty, Biology Department Ege University Izmir Türkiye

**Keywords:** fermented beverages, lactic acid bacteria, probiotic, Shalgam juice

## Abstract

The current study was meant to examine the probiotic characteristics of lactic acid bacteria (LAB) derived from commercially produced fermented black carrot (Shalgam) juices. The 48 LAB isolates were derived from samples and identified by 16S rDNA sequencing. The most prevalent strain belonged to *Pediococcus acidilactici* (56.25%), followed by *Lacticaseibacillus* sp. (20.83%), *Lentilactobacillus buchneri* (6.25%), and *L. paracasei* (6.25%). All strains demonstrated viability at low pH values except *Enterococcus faecalis* SL8. Besides, all of them exhibited resistance to bile salts (63.71%–123.46%), pepsin (42.99%–133.46%), pancreatin (63.41%–155.55%) and phenol (53.43%–177.49%) at varying levels. The hydrophobicity of the strains toward xylene varied from 1.20% to 92.61%, while the values of auto‐aggregation and co‐aggregation were in the range of 2.74%–24.91% and 1.39%–27.40%, respectively. The strains with the best probiotic characteristics ( *P. acidilactici* SL7 and SL12, *L. paracasei* SL27) were selected to be analyzed for short‐chain fatty acids production. The acetate, butyrate, and propionate production of the strains ranged from 4480 to 6480 μg/mL, 845 to 1190 μg/mL, and 15,355 to 17,450 μg/mL, respectively. These results suggest that Shalgam juice originated LAB strains could be utilized as potential probiotics in the production of fermented foods and also in pharmaceutical products.

## Graphical abstract

Investigating new probiotic cultures has become significant because of the increasing utilization of probiotics in food and pharmaceutical products. Forty‐eight LAB strains were derived from commercial Shalgam juices and identified by 16S rRNA gene sequencing in the current study. The results demonstrated that Shalgam juice could be a good probiotic source, as most of the LAB strains isolated from Shalgam juice had probiotic potential.

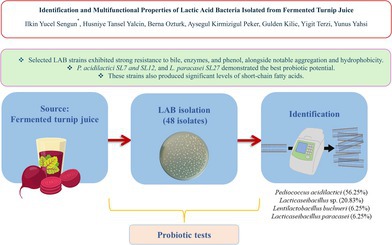

## 1 Introduction

Shalgam juice, which is a traditional Turkish fermented beverage, has a sour taste, a red‐colored and cloudy appearance (Tanguler et al. 2021). Shalgam juice, also called turnip juice, is usually produced by lactic acid fermentation using turnip, bulgur, black carrot, sourdough, salt, and water (Tanguler and Erten 2012). *Lacticaseibacillus paracasei*, *Limosilactobacillus fermentum*, *Lactiplantibacillus plantarum*, and *Levilactobacillus brevis* are the dominant species in Shalgam juice fermentation (Akman et al. 2021). Besides, Shalgam juice contains various volatile flavor compounds that contribute to aroma formation, such as volatile acids (butanoic acid, isovaleric acid, pentanoic acid, hexanoic acid, and heptanoic acid, etc.), volatile phenols, esters, higher alcohols, carbonyl compounds, and lactones.

Lactic acid bacteria (LAB) are an important group of bacteria that are commonly utilized as starter cultures in the production of fermented foods (Boricha et al. 2019). LAB provide some beneficial effects such as ensuring the microbial safety of the product, improving the organoleptic properties of the products, and extending the shelf life of food by producing metabolites such as bacteriocins, hydrogen peroxide, organic acids, diacetyl, exopolysaccharides, ethanol, aroma compounds, etc. LAB are also industrially important microorganisms due to their metabolic features such as acidification and proteolytic activities (Abbasiliasi et al. 2017). Therefore, the isolation and characterization of novel LAB strains for wider applications are of industrial importance.

Probiotics are live, non‐pathogenic microorganisms that have positive effects on the health of the host, such as regulating the immune system, preventing diarrhea, lowering cholesterol, and reducing hypertension when taken in sufficient quantities. *Lacticaseibacillus*, *Lactiplantibacillus*, *Lactococcus*, *Levilactobacillus*, *Limosilactobacillus*, *Pediococcus*, and *Streptococcus* species are among the microorganisms approved as probiotics (Akman et al. 2021). Probiotics must have certain properties to colonize and survive the gastrointestinal (GI) system.

The probiotic characteristics of LAB from fermented dairy products, fruit and vegetable products, and cereal‐based products have been screened by various researchers. However, there are few studies investigating the probiotic characteristics of strains derived from Shalgam juice to the best of the knowledge of the authors (Akman et al. 2021; Yetiman and Ortakci 2023; Sengun et al. 2024). Thus, the objectives of this research were (i) to isolate and identify strains from the samples, and (ii) to determine the probiotic properties of the strains.

## 2 Materials and Methods

### 2.1 Samples

Seven Shalgam juice samples were purchased from local markets of Adana, Hatay, and Osmaniye in Türkiye (Table 1).

**TABLE 1:** Ingredients of the Shalgam juice samples.

Shalgam juice samples	Ingredients	Sampling region
A	Turnip, black carrot, bulgur, sourdough, garlic, sodium benzoate, chili pepper, salt, water	Kırıkhan/Hatay
B	Turnip, black carrot, bulgur, salt, water	Osmaniye
C	Black carrot, bulgur, salt, water	Osmaniye
D	Black carrot, bulgur, salt, water	Osmaniye
E	Black carrot, bulgur, salt, water	Seyhan/Adana
F	Turnip, black carrot, bulgur, sourdough, garlic, sodium benzoate, salt, water	Yüreğir/Adana
G	Black carrot, bulgur, salt, water	Osmaniye

### 2.2 Isolation of Lactic Acid Bacteria

Shalgam juice (1 mL) was transferred into 9 mL peptone water (0.1%) (PW, pH 6.8 ± 0.2) and decimal dilutions were prepared, followed by plating on de Man‐Rogosa and Sharp Agar (MRS Agar, pH 5.6–5.9) and M17 Agar (pH 7.2 ± 0.2) supplemented with cycloheximide (0.1 g/L) using the double layer pour plate method. The plates were incubated at 30°C for 3–5 days (ISO 1998).

Colonies were randomly selected from MRS and M17 Agar as the square root of the colony counts. Typical colonies were tested for purity on relevant media. Catalase‐negative and Gram‐positive strains were accepted as presumptive LAB and stored at −18°C in respective media, including (15%, v/v) glycerol.

### 2.3 Identification of the Lactic Acid Bacteria

The 16S rDNA gene region was amplified using universal primers (Reverse rD1: 5′‐CGGCTACCTTGTTACGACTTC‐3′, Forward fD1: 5′‐AGAGTTTGATGGCTCA‐3′). PCR products were visualized by gel electrophoresis on (1.5%, w/v) agarose gel and subjected to Sanger sequencing. The obtained nucleotide sequences were analyzed using the Geneious software. Sequencing results were submitted to the NCBI database for further analysis. Bacterial strains were identified through a BLAST search against the NCBI database. The sequences were compared with known sequences to determine the closest taxonomic relatives. Phylogenetic analysis was conducted using MEGA 11 software, and a phylogenetic tree was constructed based on the neighbor‐joining method. The evolutionary distances were computed using the Tajima‐Nei method.

### 2.4 Assessment of Probiotic Potential

#### 2.4.1 Tolerance to Acidic pH, Bile Salt, Pepsin, Pancreatin, and Phenol

To determine the tolerance to low pH, the cultures were cultivated in MRS/M17 Broth with pH adjusted to 2, 3, and 4. After incubation at 37°C for 24 h, the formation of turbidity was interpreted as a positive result (Tambekar and Bhutada 2010).

To determine the cultures' growth in the presence of bile salt, pepsin, pancreatin, and phenol, the strains were cultivated in the respective media: (i) MRS/M17 Broth with 0.3 or 1.0% (w/v) oxgall (incubated at 37°C for 4 h) (Zielińska et al. 2015), (ii) 3 mg pepsin/mL + 0.85% NaCl, pH 2.5 (incubated at 37°C for 4 h) (Tokatlı et al. 2015), (iii) 1 mg pancreatin/mL + 0.85% NaCl + 0.3% bile salt, pH 8 (incubated at 37°C for 6 h) (Tokatlı et al. 2015), (iv) MRS/M17 Broth with 0.4% (w/v) phenol (incubated at 37°C for 24 h) (Zielińska et al. 2015), respectively. The viable cells were counted by the pour plate technique on the respective media, and the viability percentage was calculated by the following Equation 1:

(1) $$\text{Viability}\;\left(\%\right)=\left(\frac{\mathrm{Ni}}{\mathrm{Nx}}\right)*100$$

where Nx = log CFU/mL before incubation, Ni = log CFU/mL after incubation.

#### 2.4.2 Antibiotic Sensitivity

The cultures cultivated in MRS/M17 Broth were spread on MRS/M17 Agar. Antibiotic discs [tetracycline (T, 30 μg/mL), kanamycin (K, 30 μg/mL), erythromycin (E, 15 μg/mL), chloramphenicol (C, 30 μg/mL), and ampicillin (AMP, 10 μg/mL)] were placed on the plates and incubated at 37°C for 72 h. The results were stated as susceptible (S, ≥ 20 mm), intermediate resistant (I, 15–19 mm), and resistant (R, ≤ 14 mm) (Samedi and Charles 2019).

#### 2.4.3 Hemolytic Activity

The isolates were streaked on Columbia CNA Agar with sheep blood (5%) and incubated at 37°C for 48 h. The plates were interpreted for the hemolytic reaction: β‐hemolysis, α‐hemolysis, or γ‐hemolysis (Abouloifa et al. 2020).

#### 2.4.4 Antimicrobial Activity

The antimicrobial activity of cell‐free supernatant (CFS) was detected by the agar diffusion method. *Staphylococcus aureus* 6538P, *Salmonella typhimurium* NRRL B‐4420, *Listeria monocytogenes* Scott A, *Escherichia coli* O157:H7 ATCC 43895, *E. coli* ATCC 1103, *Enterococcus faecalis* ATCC 29212, and *Bacillus subtilis* ATCC 6037 were used as indicator cultures. One hundred microliters of inoculum was added to Plate Count Agar (20 mL) (PCA, pH 7.2 ± 0.2), rapidly poured into plates, and kept at 25°C for 20 min. After that, wells with a 6 mm diameter were drilled by a sterile cork porer. CFS (100 μL) was added to the wells. The plates were incubated at 37°C for 24 h, and the inhibition zones (mm) were measured (including well diameter) (Akman et al. 2021).

#### 2.4.5 Tolerance to NaCl

The isolates were cultivated in MRS/M17 Broth supplemented with NaCl (1.5 and 10%, w/v). After the incubation period (at 30°C for 24 h), the formation of turbidity was evaluated as a positive result (Samedi and Charles 2019).

#### 2.4.6 Enzymatic Activity

##### 2.4.6.1 Proteolytic Activity

To determine the proteolytic activity of the strains, the cultures were transferred to Skim Milk Medium (10%, v/v) and incubated at 30°C for 42 h. Subsequently, 1 mL of distilled water and 10 mL of 0.72 N trichloroacetic acid were added to the samples and held for 10 min. Ten mL of Na_2_CO_3_‐Na_4_P_2_O_7_ solution and 3 mL of phenol reagent were added to 5 mL of filtered samples and stirred until the blue color was formed. The blue color samples were centrifuged at 4500 rpm at 4°C for 15 min, and the clear blue solution was measured at 650 nm. The results were stated as mg/mL tyrosine (Şimşek et al. 2006).

##### 2.4.6.2 β‐Galactosidase Activity

The cultures were transferred into MRS (containing glucose) and M17 Broth. Ortho Nitrophenyl‐β‐D‐Galactopyronoside peptone medium (0.25 mL) was added to the suspension, prepared such that a loop of inoculum was suspended in physiological saline (0.25 mL). The solution was incubated at 30°C for 3–4 h (Hébert et al. 2000). According to the intensity of yellow color formation (by a spectrophotometer at 420 nm), the outcomes were evaluated as very high (++++), high (+++), middle (++), or low (+).

#### 2.4.7 In Vitro Adhesion Ability

For auto‐aggregation assays, the overnight cultures were centrifuged at 4000x g at 4°C for 10 min. The pellet was then suspended in PBS to obtain an OD of 0.25 at 600 nm. The suspension (0.3 mL) was vortexed for 10 s and incubated at 37°C for 4 h. The absorbance of the upper suspension was measured at 600 nm by spectrophotometer for 0 h and 4 h (Gil‐Rodríguez et al. 2015). The auto‐aggregation ability of strains was determined using Equation 2.

(2) $$\text{Auto}-\text{aggregation}\;\left(\%\right)=\left[1-\frac{\mathrm{At}}{A0}\right]*100$$

where At = absorbance for 4 h, A0 = absorbance for 0 h.

The co‐aggregation ability of the strains with *S. typhimurium* NRRL B‐4420 was also examined. The initial measurement of absorbance was conducted for both the LAB (*A*_LAB_) and pathogen (*A*_P_) suspensions at a wavelength of 600 nm. Subsequently, equal volumes of each cell suspension (1.5 mL) were mixed and vortexed for 10 s. After 4 h, the absorbance at 600 nm was measured (Gil‐Rodríguez et al. 2015). Equation 3 was used to calculate the percentage of co‐aggregation.

(3) $$\mathrm{Co}-\text{aggregation}\;\left(\%\right)=\left[\frac{\frac{\left(A_{\mathrm{LAB}}+A_{P}\right)}{2}-\left(A_{\mathrm{mix}}\right)}{\frac{\left(A_{\mathrm{LAB}}+A_{P}\right)}{2}}\right]\times 100$$

where *A*_P_ = initial absorbance of pathogen, *A*_LAB_ = initial absorbance of LAB, *A*_mix_ = absorbance of mixture for 4 h.

To determine the cell surface hydrophobicity of the strains, the isolates were centrifuged at 4500 rpm at 4°C for 15 min, and the pellet was suspended with PBS (OD 1.0) at 600 nm using a spectrophotometer. Xylene (1.5 mL) was mixed with an equal volume of cell suspension for 120 s. The absorbance of the aqueous phase was measured at 600 nm (Syal and Vohra 2013). The percentage of hydrophobicity was calculated using the following Equation 2, where At = absorbance after extraction with xylene, A0 = absorbance before extraction.

#### 2.4.8 Production of Short‐Chain Fatty Acids

The strains activated in the relevant medium (1% yeast extract, 2% peptone, and 2% inulin) were incubated at 37°C for 18 h under anaerobic conditions. The CFS, which was obtained by centrifugation (at 4800 ***g*** for 5 min), was filtered (0.45 μm, Sartorius Stedim). The amounts of SCFAs were determined by HPLC Agilent 1260 Infinity, DAD detector equipped with an Agilent C8 column (150 × 4.6 mm with 5 μm). The mobile phase was used as 10 mM H_2_SO_4_ (100%) at a flow rate of 0.8 mL/min. The chromatogram was assayed at 210 nm (Pabari et al. 2020).

#### 2.4.9 Statistical Analysis

The data from the assays were subjected to one‐way ANOVA at the 0.05 level of significance using the software package SPSS 25.0 and are presented as mean ± standard deviation (SPSS 2020).

## 3 Results

### 3.1 Microbiological Properties of Shalgam Juices

In the present study, LAB counts were determined in seven different Shalgam juice samples using two media (MRS and M17 Agar). The LAB counts on MRS Agar ranged from 3.38 to 6.03 log CFU/mL, except for G, which is lower than 1 log CFU/mL (Table 2). Besides, the LAB counts in Shalgam juice samples (A, B, C and F) on M17 Agar were 1.55, 1.98, 3.62, and 3.97 log CFU/mL, respectively, while the counts in D, E, and G on M17 Agar were under the detection limit (< 1 log CFU/mL) (*p* < 0.05) (Table 2).

**TABLE 2:** LAB counts of Shalgam juice samples.

Shalgam juice samples	LAB counts (log CFU/mL)	
	MRS	M17
A	3.38 ± 0.29 b,A	1.55 ± 0.08 b,B
B	5.18 ± 0.14 e,A	1.98 ± 0.04 c,B
C	6.03 ± 0.12 f,A	3.62 ± 0.31 d,B
D	4.58 ± 0.03 d,A	< 1 a,B
E	3.98 ± 0.13 c,A	< 1 a,B
F	5.05 ± 0.10 e,A	3.97 ± 0.15 e,B
G	< 1a	< 1a

*Note:* Values in the same column with different lower cases (a, b, c, d, e, f) and in the same row with different upper cases (A, B) are significantly different (*p* < 0.05).

### 3.2 Isolation and Identification of Lactic Acid Bacteria Isolates

**FIGURE 1:**
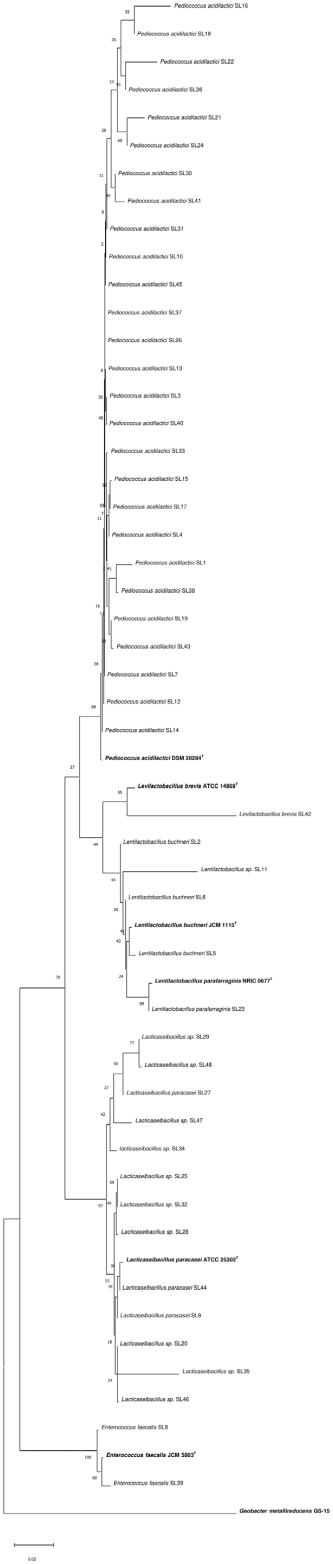
Phylogenetic relationships of lactic acid bacteria isolates from the Shalgam juice samples.

In the current study, microorganisms derived from Shalgam juice samples were identified to reveal the diversity of LAB. Catalase‐negative and Gram‐positive isolates growing in MRS and M17 Agar (48 strains) were evaluated as potential LAB. As a result of the isolation, 48 bacterial strains were obtained. According to the identification results, the bacterial strains belonged to the genera *Lacticaseibacillus*, *Lentilactobacillus*, *Pediococcus*, *Levilactobacillus*, and *Enterococcus*. The genera and species are summarized in Table 3. As the 16S rRNA gene region was insufficient for identifying some isolates, some bacterial strains were characterized at the genus level. In the phylogenetic tree, similar species and genera cluster in specific, close branches, confirming the performed identification (Figure 1).

**TABLE 3:** Genotypic identification results of LAB isolates.

Strain	Source of isolates	Medium of isolation	Identification (Sanger method)	Accession number in NCBI	Identify (%)	PCR‐product length (bp)
SL1	A	MRS	*Pediococcus acidilactici*	OR622973	98.39	558
SL2	A	MRS	*Lentilactobacillus buchneri*	OR622967	100.00	560
SL3	A	MRS	*Pediococcus acidilactici*	OR622974	99.46	744
SL4	A	MRS	*Pediococcus acidilactici*	OR622975	100.00	587
SL5	A	MRS	*Lentilactobacillus buchneri*	OR622968	99.85	661
SL6	A	MRS	*Lentilactobacillus buchneri*	OR622969	100.00	659
SL7	A	MRS	*Pediococcus acidilactici*	OR622976	100.00	572
SL8	A	M17	*Enterococcus faecalis*	OR622952	100.00	757
SL9	A	M17	*Lacticaseibacillus paracasei*	OR622954	100.00	661
SL10	A	M17	*Pediococcus acidilactici*	OR622977	100.00	584
SL11	A	M17	*Lentilactobacillus* sp.	OR622971	95.88	490
SL12	B	MRS	*Pediococcus acidilactici*	OR622978	99.73	736
SL13	B	MRS	*Pediococcus acidilactici*	OR622979	100.00	675
SL14	B	MRS	*Pediococcus acidilactici*	OR622980	100.00	643
SL15	B	MRS	*Pediococcus acidilactici*	OR622981	99.13	693
SL16	B	MRS	*Pediococcus acidilactici*	OR622982	95.67	536
SL17	B	MRS	*Pediococcus acidilactici*	OR622983	99.84	633
SL18	B	MRS	*Pediococcus acidilactici*	OR622984	99.73	365
SL19	C	MRS	*Pediococcus acidilactici*	OR622985	100.00	598
SL20	C	MRS	*Lacticaseibacillus* sp.	OR622958	100.00	536
SL21	C	MRS	*Pediococcus acidilactici*	OR622986	97.97	640
SL22	C	MRS	*Pediococcus acidilactici*	OR622987	96.82	501
SL23	C	MRS	*Lentilactobacillus parafarraginis*	OR622970	100.00	636
SL24	C	MRS	*Pediococcus acidilactici*	OR622988	100.00	339
SL25	C	MRS	*Lacticaseibacillus* sp.	OR622959	99.69	643
SL26	D	MRS	*Pediococcus acidilactici*	OR622989	100.00	671
SL27	D	MRS	*Lacticaseibacillus paracasei*	OR622955	98.63	438
SL28	D	MRS	*Lacticaseibacillus* sp.	OR622960	99.84	609
SL29	D	MRS	*Lacticaseibacillus* sp.	OR622961	98.66	449
SL30	D	MRS	*Pediococcus acidilactici*	OR622990	98.39	559
SL31	D	MRS	*Pediococcus acidilactici*	OR622991	100.00	578
SL32	E	MRS	*Lacticaseibacillus* sp.	OR622962	99.86	698
SL33	E	MRS	*Pediococcus acidilactici*	OR622992	99.34	602
SL34	E	MRS	*Lacticaseibacillus* sp.	OR622957	99.36	316
SL35	E	MRS	*Lacticaseibacillus* sp.	OR622963	96.49	627
SL36	E	MRS	*Pediococcus acidilactici*	OR622993	99.08	326
SL37	E	MRS	*Pediococcus acidilactici*	OR622994	100.00	635
SL38	E	MRS	*Pediococcus acidilactici*	OR622995	99.50	604
SL39	F	MRS	*Enterococcus faecalis*	OR622953	99.46	555
SL40	F	MRS	*Pediococcus acidilactici*	OR622996	100.00	675
SL41	F	MRS	*Pediococcus acidilactici*	OR622997	98.46	712
SL42	F	MRS	*Levilactobacillus brevis*	OR622972	94.55	256
SL43	F	MRS	*Pediococcus acidilactici*	OR622998	99.69	646
SL44	F	M17	*Lacticaseibacillus paracasei*	OR622956	100.00	652
SL45	F	M17	*Pediococcus acidilactici*	OR622999	99.82	563
SL46	F	M17	*Lacticaseibacillus* sp.	OR622964	100.00	548
SL47	F	M17	*Lacticaseibacillus* sp.	OR622966	98.21	337
SL48	F	M17	*Lacticaseibacillus* sp.	OR622965	98.19	197

### 3.3 Probiotic Potential of Lactic Acid Bacteria Strains

#### 3.3.1 Stress Tolerance of the Strains

In the current study, the tolerance of the strains to stress conditions such as acidic pH and the presence of bile salt, pepsin, pancreatin, and phenol was examined. The results of the study demonstrated that all LAB strains remained alive after exposure to pH 2, 3, and 4, except *E. faecalis* SL8 (Table 4). Besides, the strains survived in 0.3% and 1% bile salts in the range of 66.40%–122.58% and 63.71%–123.46%, respectively (Table 4). *P. acidilactici* SL26 and *P. acidilactici* SL43 demonstrated the highest tolerance to 0.3% and 1.0% bile salts, respectively. LAB strains showed viability rates ranging from 42.99% to 133.46% in simulated gastric juice for 4 h, while survival rates ranged between 63.41% and 155.55% in artificial intestinal juice for 6 h (Table 4). *Lacticaseibacillus* sp. SL20 and *P. acidilactici* SL24 exhibited higher resistance to simulated gastric juice with viability rates of 121.96% and 133.46%, respectively. *P. acidilactici* SL36 demonstrated the highest resistance to pancreatin‐containing medium, followed by *P. acidilactici* SL7 (117.86%) and *P. acidilactici* SL37 (116.79%). The results also demonstrated that LAB strains survived rates in the range of 53.43%–177.49% in phenol (Table 4). *P. acidilactici* SL22 and *L. paracasei* SL27 showed quite high resistance to phenol with viability rates of 177.49% and 170.31%, respectively.

**TABLE 4:** Viability of LAB strains under stress conditions.

Strains				Viability (%)				
	pH			Bile tolerance		Pepsin	Pancreatin	Phenol
	2	3	4	0.3%	1.0%			0.4%
SL1	+	+	+	98.29	107.00	94.84	104.17	105.19
SL2	+	+	+	90.01	99.01	84.31	110.67	99.56
SL3	+	+	+	96.29	92.58	85.28	111.67	104.72
SL4	+	+	+	118.03	107.46	100.52	105.97	90.36
SL5	+	+	+	104.09	119.50	92.17	100.67	96.26
SL6	+	+	+	110.37	118.62	98.04	102.42	99.91
SL7	+	+	+	97.24	106.72	100.85	117.86	98.02
SL8	−	−	+	91.55	122.94	96.30	100.50	107.83
SL9	+	+	+	108.75	108.67	72.97	111.10	86.34
SL10	+	+	+	100.85	106.50	62.59	107.69	76.98
SL11	+	+	+	117.11	117.05	56.91	113.15	88.09
SL12	+	+	+	108.63	107.94	99.75	63.41	102.82
SL13	+	+	+	106.56	98.50	95.18	112.63	70.53
SL14	+	+	+	93.51	104.42	93.73	96.60	107.70
SL15	+	+	+	104.71	110.32	81.40	103.10	94.12
SL16	+	+	+	91.88	113.33	100.37	107.45	78.57
SL17	+	+	+	86.12	77.61	97.99	92.98	101.20
SL18	+	+	+	110.60	122.03	91.19	98.93	72.95
SL19	+	+	+	110.15	100.00	102.46	101.35	100.70
SL20	+	+	+	84.17	112.62	121.96	111.52	74.93
SL21	+	+	+	107.54	112.56	87.89	101.04	85.60
SL22	+	+	+	97.25	96.83	115.93	108.51	177.49
SL23	+	+	+	92.51	87.31	101.33	97.84	63.31
SL24	+	+	+	105.82	100.49	133.46	91.74	101.80
SL25	+	+	+	104.80	98.22	106.65	107.75	100.12
SL26	+	+	+	122.58	116.04	115.16	108.59	104.73
SL27	+	+	+	109.79	98.42	103.08	108.68	170.31
SL28	+	+	+	89.49	115.87	97.82	83.43	111.62
SL29	+	+	+	109.91	109.65	96.85	100.33	85.22
SL30	+	+	+	119.34	109.06	102.36	114.05	90.35
SL31	+	+	+	110.09	101.89	99.08	99.23	85.08
SL32	+	+	+	103.68	123.34	117.05	103.99	94.54
SL33	+	+	+	94.98	103.43	88.93	102.97	84.20
SL34	+	+	+	109.49	117.27	96.34	107.35	99.52
SL35	+	+	+	66.40	63.71	114.37	103.35	53.43
SL36	+	+	+	68.70	97.23	110.53	155.55	84.70
SL37	+	+	+	106.13	105.43	113.46	116.79	86.28
SL38	+	+	+	97.92	106.28	117.16	110.35	81.44
SL39	+	+	+	119.19	106.83	109.26	102.86	117.51
SL40	+	+	+	101.96	99.28	108.52	98.95	90.22
SL41	+	+	+	104.72	104.23	107.89	102.43	87.48
SL42	+	+	+	103.35	102.00	113.05	101.26	108.12
SL43	+	+	+	104.51	123.46	101.88	104.51	106.30
SL44	+	+	+	97.10	104.65	43.96	107.38	81.90
SL45	+	+	+	89.17	104.48	51.07	93.54	91.18
SL46	+	+	+	95.97	91.41	54.86	109.32	82.52
SL47	+	+	+	115.88	102.75	42.99	96.39	87.98
SL48	+	+	+	91.05	101.13	48.14	103.84	90.72

#### 3.3.2 Biosafety Properties of LAB Strains

All tested strains demonstrated resistance to kanamycin except *Lacticaseibacillus* sp. SL29 (S) and SL46 (I). However, almost all strains were susceptible to ampicillin, chloramphenicol, and erythromycin. A variable susceptibility was observed to tetracycline depending on the LAB strains (Table 5). Besides, *L. paracasei* SL27 was the only isolate to show resistance to all antibiotics used. The results of the current study also showed that all LAB indicated γ hemolysis (Table 5).

**TABLE 5:** Biosafety properties of the LAB strains.

Strains	Antibiotic resistance (mm)					Hemolytic activity
	AMP (10 μg/mL)	C (30 μg/mL)	E (15 μg/mL)	K (30 μg/mL)	T (30 μg/mL)	
SL1	S	S	S	R	S	γ
SL2	S	S	S	R	S	γ
SL3	S	S	S	R	I	γ
SL4	S	S	S	R	S	γ
SL5	S	S	S	R	S	γ
SL6	S	S	S	R	S	γ
SL7	S	S	S	R	R	γ
SL8	S	S	I	R	I	γ
SL9	R	I	R	R	S	γ
SL10	S	S	I	R	R	γ
SL11	I	S	R	R	R	γ
SL12	S	S	S	R	R	γ
SL13	S	S	S	R	R	γ
SL14	S	S	S	R	R	γ
SL15	S	S	S	R	I	γ
SL16	S	S	S	R	R	γ
SL17	S	S	S	R	S	γ
SL18	S	S	S	R	I	γ
SL19	S	S	S	R	R	γ
SL20	S	S	S	R	R	γ
SL21	I	S	S	R	S	γ
SL22	S	S	S	R	R	γ
SL23	S	S	S	R	R	γ
SL24	S	S	S	R	I	γ
SL25	S	S	S	R	R	γ
SL26	S	S	I	R	I	γ
SL27	R	R	R	R	R	γ
SL28	S	S	S	R	R	γ
SL29	S	S	S	S	I	γ
SL30	S	S	S	R	I	γ
SL31	S	S	S	R	I	γ
SL32	S	S	S	R	S	γ
SL33	S	S	S	R	R	γ
SL34	S	S	S	R	I	γ
SL35	S	S	S	R	R	γ
SL36	I	S	I	R	S	γ
SL37	S	S	S	R	R	γ
SL38	S	S	S	R	I	γ
SL39	S	S	S	R	I	γ
SL40	S	S	S	R	I	γ
SL41	S	S	S	R	R	γ
SL42	S	S	S	R	R	γ
SL43	S	S	S	R	R	γ
SL44	S	S	S	R	S	γ
SL45	S	R	R	R	I	γ
SL46	S	S	R	I	S	γ
SL47	S	S	S	R	R	γ
SL48	S	S	S	R	R	γ

Abbreviations: I, Intermediate; R, Resistant; S, Sensitive.

#### 3.3.3 Antimicrobial Activity of LAB Strains

LAB strains demonstrated inhibition zones between 7 and 26 mm against microorganisms (Table 6). Fifteen strains indicated antimicrobial activity against all microorganisms ( *S. typhimurium* 11–19 mm, *S. aureus* 11–24 mm, *L. monocytogenes* 10–26 mm, *E. faecalis* 11–24 mm, *E. coli* O57:H7 10–25 mm, *E. coli* 10–19 mm, and *B. subtilis* 10–20 mm). However, four strains (SL11, SL44, SL45, and SL48) showed no antibacterial activity on the test cultures. The highest inhibition zone was developed by *P. acidilactici* SL36 against *L. monocytogenes* (26 mm). *E. coli* O157:H7 was found to be the most sensitive microorganism to LAB supernatants.

**TABLE 6:** Clear zone of inhibition of LAB strains (well diameter: 6 mm).

Strains	Antimicrobial activity (mm)						
	*B. subtilis*	*E. coli*	*E. coli* O157:H7	*E. faecalis*	*L. monocytogenes*	*S. aureus*	*S. typhimurium*
SL1	12	6	20	12	18	25	12
SL2	8	6	21	12	17	8	12
SL3	12	6	25	13	17	7	12
SL4	12	6	19	14	16	10	15
SL5	15	6	23	13	16	15	15
SL6	6	6	24	13	22	8	12
SL7	14	11	25	17	25	19	16
SL8	6	6	6	8	6	6	6
SL9	6	6	6	6	10	6	6
SL10	6	6	6	10	6	6	6
SL11	6	6	6	6	6	6	6
SL12	9	12	12	10	6	6	11
SL13	6	10	12	6	6	6	11
SL14	16	10	24	17	21	12	16
SL15	10	6	24	17	20	12	13
SL16	6	6	12	6	6	6	11
SL17	6	8	12	9	11	9	10
SL18	15	6	24	15	23	9	15
SL19	10	16	21	14	13	14	19
SL20	15	17	15	15	17	12	13
SL21	16	15	14	14	13	13	13
SL22	17	16	12	13	12	11	13
SL23	15	15	17	13	14	13	12
SL24	11	12	18	11	13	13	12
SL25	18	14	18	14	12	12	13
SL26	6	10	12	8	11	12	10
SL27	12	14	12	11	17	15	12
SL28	14	16	25	24	25	24	14
SL29	20	10	21	20	22	19	15
SL30	6	15	14	9	12	16	11
SL31	6	12	10	9	9	14	9
SL32	6	6	18	6	6	6	15
SL33	6	6	13	6	6	6	6
SL34	6	6	11	6	6	6	6
SL35	8	19	22	21	20	15	19
SL36	20	18	25	23	26	18	18
SL37	6	6	12	6	6	6	6
SL38	6	6	20	6	6	20	10
SL39	6	6	12	6	6	6	12
SL40	10	13	13	12	10	12	11
SL41	6	13	15	10	6	12	11
SL42	10	14	13	10	6	15	12
SL43	6	6	13	6	10	6	12
SL44	6	6	6	6	6	6	6
SL45	6	6	6	6	6	6	6
SL46	6	6	6	8	6	6	6
SL47	6	6	6	7	6	6	6
SL48	6	6	6	6	6	6	6

*Note:* Well diameter: 6 mm.

#### 3.3.4 Assessment of Technological Properties

The technological properties of the isolates were evaluated in terms of salt concentrations, proteolytic, and β‐galactosidase enzyme activity. All LAB strains showed viability at 1.5% salt, while only 12 isolates were viable at 10% salt (Table 7). The results of the study showed that the proteolytic enzyme activity of the strains ranged from 0.004 to 0.095 mg tyrosine/mL (Table 7). Hence, the proteolytic enzyme activity of six strains (SL9, SL28, SL31, SL34, SL37, and SL38) was higher than 0.050 mg tyrosine/mL, while four isolates (SL6, SL28, SL32, and SL33) showed no proteolytic enzyme activity. In the present study, *L. parafarraginis* SL23, *P. acidilactici* SL21, SL22, and SL24 indicated very high levels of β‐galactosidase enzyme activity. However, 17 strains showed no β‐galactosidase enzyme activity (Table 7).

**TABLE 7:** Technological properties of LAB strains.

Strains	NaCl		Proteolytic activity (mg tyrosine/mL)	β‐Galactosidase activity
	1.5%	10%		
SL1	+	−	0.027	−
SL2	+	−	0.023	−
SL3	+	−	0.013	+
SL4	+	−	0.021	+++
SL5	+	−	0.022	+++
SL6	+	−	−	−
SL7	+	−	0.013	+
SL8	+	+	0.030	−
SL9	+	−	0.051	−
SL10	+	−	0.035	−
SL11	+	−	0.020	−
SL12	+	−	0.021	−
SL13	+	−	0.021	+
SL14	+	−	0.015	−
SL15	+	+	0.016	−
SL16	+	−	0.010	+
SL17	+	−	0.017	−
SL18	+	−	0.022	−
SL19	+	−	0.045	+
SL20	+	+	0.017	+++
SL21	+	−	0.037	++++
SL22	+	+	0.012	++++
SL23	+	+	0.013	++++
SL24	+	−	0.022	++++
SL25	+	−	0.017	+++
SL26	+	−	0.004	+++
SL27	+	+	0.019	−
SL28	+	+	−	−
SL29	+	+	0.021	+
SL30	+	−	0.014	+
SL31	+	−	0.075	+
SL32	+	+	−	+
SL33	+	−	−	−
SL34	+	−	0.005	+
SL35	+	−	0.095	−
SL36	+	+	0.014	−
SL37	+	+	0.022	+
SL38	+	+	0.005	++
SL39	+	−	0.058	++
SL40	+	−	0.031	++
SL41	+	−	0.028	++
SL42	+	−	0.038	++
SL43	+	−	0.040	+++
SL44	+	−	0.059	+++
SL45	+	−	0.075	+++
SL46	+	−	0.044	+++
SL47	+	−	0.049	++
SL48	+	−	0.036	+

*Note:* (++++) very high, (+++) high, (++) middle, (+) low.

#### 3.3.5 Cell Surface Properties

In the present study, LAB strains were subjected to auto‐aggregation, co‐aggregation, and cell surface hydrophobicity assays to evaluate their adhesion ability to the intestinal mucosa. In this study, the auto‐aggregation ability of LAB strains ranged from 2.84% to 24.91% (Table 8). The highest auto‐aggregation was exhibited by *L. buchneri* SL6 (24.91%), followed by *P. acidilactici* SL17 (23.58%) and SL1 (22.17%). Additionally, the strains demonstrated co‐aggregation ability with *S. typhimurium* , ranging from 1.39% to 27.40% (Table 8). *P. acidilactici* SL45 (27.40%) indicated the highest co‐aggregation ability, which was followed by *L. buchneri* SL6 (26.02%). In the current study, the cell surface hydrophobicities of the strains were also examined to determine their adhesion ability to the intestinal mucosa. The hydrophobicity values of all strains were between 1.20% and 92.61% toward xylene, except for SL19, SL28, SL29, SL34, and SL38 (Table 8). *Lacticaseibacillus* sp. SL48 was less adherent (1.20%) to xylene. The highest hydrophobicity was exhibited by *P. acidilactici* SL18 (92.61%), which was followed by *P. acidilactici* SL34 (89.08%).

**TABLE 8:** Cell surface properties of LAB strains.

Strains	Auto‐aggregation (%)	Co‐aggregation (%)	Hydrophobicity (%)
SL1	22.17 ± 0.10 u	13.78 ± 0.96 ij	25.08 ± 0.13 kl
SL2	6.21 ± 0.30 defg	15.17 ± 0.38 j	56.12 ± 0.08 ç
SL3	17.55 ± 0.32 s	13.06 ± 0.25 i	32.07 ± 0.20 p
SL4	8.64 ± 0.70 def	3.48 ± 0.28 cd	30.59 ± 0.13 n
SL5	— a	7.23 ± 0.68 ef	13.81 ± 0.26 f
SL6	24.91 ± 0.21 v	26.02 ± 0.18 pr	12.42 ± 0.19 e
SL7	7.84 ± 1.46 ghijk	24.70 ± 1.10 op	43.86 ± 0.08 y
SL8	6.76 ± 0.30 efghi	1.39 ± 0.70 ab	40.24 ± 0.21 ü
SL9	5.08 ± 0.19 cde	2.38 ± 1.25 bcd	77.99 ± 0.05 ı
SL10	2.94 ± 0.26 b	3.54 ± 2.06 cd	31.84 ± 0.33 p
SL11	— a	— a	23.26 ± 0.34 j
SL12	— a	8.78 ± 0.05 fgh	28.51 ± 0.31 m
SL13	7.02 ± 0.40 efghij	12.80 ± 0.50 i	25.46 ± 0.08 l
SL14	11.90 ± 0.30 mn	19.49 ± 0.15 lm	35.63 ± 0.15 s
SL15	4.39 ± 0.80 bcd	25.26 ± 0.27 p	20.86 ± 0.32 h
SL16	3.22 ± 1.51 bc	— a	33.79 ± 0.23 r
SL17	23.58 ± 1.82 uv	12.70 ± 1.53 i	8.10 ± 0.08 c
SL18	12.40 ± 1.12 no	19.12 ± 0.64 klm	92.61 ± 0.02 ş
SL19	6.59 ± 0.01 efgh	7.21 ± 0.21 ef	— a
SL20	7.67 ± 0.08 ghijk	2.76 ± 0.12 bcd	24.38 ± 0.21 k
SL21	8.63 ± 0.32 hijkl	12.58 ± 0.01 i	28.89 ± 0.15 m
SL22	7.32 ± 0.37 fghijk	8.65 ± 0.10 fgh	53.09 ± 0.14 x
SL23	8.36 ± 0.14 ghijkl	9.28 ± 0.40 gh	48.11 ± 0.10 w
SL24	12.90 ± 0.13 nop	1.97 ± 0.16 abc	40.69 ± 0.10 üv
SL25	9.02 ± 0.07 jkl	10.05 ± 0.27 h	33.71 ± 0.27 r
SL26	8.57 ± 0.56 hijkl	— a	43.39 ± 0.13 y
SL27	11.93 ± 0.49 mn	2.82 ± 0.33 bcd	35.25 ± 0.23 s
SL28	20.07 ± 0.45 t	23.09 ± 0.23 no	— a
SL29	15.88 ± 0.07 rs	4.06 ± 1.04 d	— a
SL30	16.63 ± 0.03 s	2.48 ± 0.30 bcd	89.08 ± 0.02 o
SL31	8.82 ± 0.81 ijkl	7.70 ± 0.37 efg	34.17 ± 0.27 r
SL32	5.41 ± 0.12 def	6.31 ± 0.52 e	41.41 ± 0.24 v
SL33	8.12 ± 1.23 ghijkl	15.50 ± 1.24 j	15.34 ± 0.17 g
SL34	2.74 ± 0.36 b	— a	— a
SL35	11.85 ± 0.25 mn	7.92 ± 0.20 efg	55.50 ± 0.12 ç
SL36	11.54 ± 0.13 mn	12.76 ± 0.27 i	28.77 ± 0.10 m
SL37	8.04 ± 0.07 ghijk	22.75 ± 0.40 n	20.50 ± 0.12 h
SL38	6.88 ± 0.28 efghij	27.40 ± 0.77 r	— a
SL39	14.02 ± 1.74 opr	20.90 ± 0.45 m	10.44 ± 0.19 d
SL40	14.51 ± 0.35 pr	18.22 ± 0.97 kl	40.10 ± 0.19 ü
SL41	12.48 ± 0.34 nop	— a	41.58 ± 0.09 v
SL42	10.27 ± 1.00 lm	4.17 ± 0.30 d	46.93 ± 0.10 q
SL43	11.95 ± 0.10 mn	17.58 ± 0.11 k	37.29 ± 0.14 t
SL44	5.24 ± 0.86 def	— a	37.93 ± 0.30 t
SL45	14.17 ± 0.25 opr	— a	22.32 ± 0.54 i
SL46	8.76 ± 0.41 hijkl	— a	44.93 ± 0.27 z
SL47	9.34 ± 0.28 kl	— a	37.46 ± 0.31 t
SL48	2.84 ± 0.63 b	— a	1.20 ± 0.19 b

*Note:* Values in the same column with different lower cases are significantly different (*P* < 0.05).

#### 3.3.6 Production of Short‐Chain Fatty Acids

The acetate, butyrate, and propionate production of the strains ranged from 4480 to 6480 μg/mL, 845 to 1190 μg/mL, and 15,355 to 17,450 μg/mL, respectively (Table 9). *P. acidilactici* SL7 and *P. acidilactici* SL12 demonstrated the highest acetate, butyrate, and propionate production, respectively. The acetate and propionate production of all strains was found to be lower compared to the control, while butyrate production was determined to be quite higher than the control (*p* < 0.05).

**TABLE 9:** Production of short‐chain fatty acids of LAB strains.

Strains	Short‐chain fatty acids (μg/mL)		
	Acetate	Butyrate	Propionate
SL7	6480 ± 155 c	965 ± 35 c	17,430 ± 42 b
SL12	5120 ± 14 b	845 ± 77 b	17,450 ± 0 b
SL27	4480 ± 70 a	1190 ± 42 d	15,355 ± 388 a
Probiotic mix	13,250 ± 56 d	0.119 ± 0.002 a	21,164 ± 36 c

*Note:* Values in the same column with different lower case (a, b, c, d) are significantly different (*P* < 0.05).

## 4 Discussion

The resistance of LAB strains to low pH values is one of the main criteria in the selection of probiotic bacteria (Behbahani et al. 2024). Similar to our results, in a study performed by Akman et al. (2021), ten different LAB ( *L. plantarum* , *L. pentosus* , and *L. fermentum* ) obtained from Gilaburu and Shalgam juices were reported to survive at pH 2.5. In another study, LAB strains ( *E. faecalis* , *L. plantarum* , *L. paraplantarum* , and *Weissella paramesenteroides* ) isolated from the surface of fresh leaves (papaya, yam, taro, sugarcane, and cassava) showed high resistance to pH 2 and 3 (Samedi and Charles 2019). The results of the studies revealed that LAB strains isolated from acidic environments could withstand low pH values, which show the capability of these strains to survive in the stomach before reaching the small intestine. The inability of probiotics to survive in acidic conditions occurs when DNA and proteins are damaged due to the decreased intracellular pH, and the activity of H^+^‐ATPase, an enzyme accountable for the regulation of the internal and external hydrogen concentrations decreases (Barzegar et al. 2023).

Bile salts, which are produced in the liver and are toxic to living cells, disrupt the structure of the cell membrane. Therefore, probiotics must be resistant to bile salts to colonize the digestive tract (Behbahani et al. 2024). Similar to our results, Akman et al. (2021) stated that LAB strains isolated from Shalgam juice were tolerant to bile salts (0.3%, 0.5%, 1%, and 1.5%) with survival rates between 89.94% and 107.82%. In another study, LAB strains ( *L. harbinensis L. plantarum* , and *L. satsumensis* ) derived from water kefir, water kefir grain, and Braga survived in 0.8% and 2% bile salts for 3 h in the range of 45%–66% (Angelescu et al. 2019). Wu et al. (2021) reported that *L. fermentum* , *L. paracasei* , and *L. parafarraginis* strains isolated from Costa Rican pineapple silages demonstrated viability rates below 50% in 0.3% bile salt. These results indicated that the resistance of LAB to bile salts may vary depending on the strain and bile salt concentration. The resistance of probiotic strains to bile salts is related to the bubble salt hydrolase enzyme, which hydrolyzes conjugated bile salts, reducing their toxic effects (Rahmati‐Joneidabad et al. 2024).

Pepsin and pancreatin resistance are also important factors in the survival of LAB strains in GI conditions. Our results are mostly higher than those reported in the literature. Angelescu et al. (2019) reported that *L. ghanensis* , *L. harbinensis* , *L. plantarum* , and *L. satsumensis* derived from fermented beverages showed survival rates between 40%–90% and 89%–99% in pepsin and pancreatin, respectively. In another study, *L. plantarum* isolated from fermented rice beverage tolerated simulated gastric and intestinal juices with viability rates of 57.29% and 83.62%, respectively (Das et al. 2020).

Gut bacteria deaminate aromatic amino acids derived from dietary proteins, resulting in the formation of phenol (Behbahani et al. 2024c). Thus, resistance to phenol with bacteriostatic activity is an important factor for LAB strains to survive under GI conditions. Our results are higher than the results obtained by Abbasiliasi et al. (2017), who reported that *P. acidilactici* Kp10 derived from traditional dried curd showed resistance to 0.4% phenol, with a viability rate of 68.64%. In another study, *L. pentosus* CHIG, *L. pentosus* NAG1, and *L. fermentum* PRS1 isolated from fermented chilly, naspati, and peru demonstrated 90.51%, 107.36%, and 87.43% viability in the presence of 0.4% phenol, respectively (Boricha et al. 2019).

According to the “Qualified Safety Assumption” concept developed by the EFSA, antibiotic resistance of LAB is an essential safety criterion, as LAB strains can transfer antibiotic resistance genes on mobile to pathogens. Similar to our results, in a study conducted by Sornsenee et al. (2021), eight different *L. paracasei* strains derived from fermented palm sap showed resistance to kanamycin but susceptibility to tetracycline, erythromycin, chloramphenicol, and ampicillin. Samedi and Charles (2019) also reported that *E. faecalis* , *L. paraplantarum* , *L. plantarum* , *W. paramesenteroides* C04, and Y05 isolated from sugarcane, papaya, taro, cassava, and yam were susceptible to ampicillin, chloramphenicol, and tetracycline. In another study, *L. paracasei* derived from yogurt exhibited resistance against ampicillin, chloramphenicol, ciprofloxacin, erythromycin, imipenem, nalidixic acid, and nitrofurantoin (Behbahani et al. 2024b). These studies have shown that the antibiotic resistance of LAB varies depending on the strain and the antibiotics used. Non‐pathogenicity of LAB strains is an essential criterion in the selection of probiotic cultures (Saboktakin‐Rizi et al. 2021). Similar to our results, many studies reported that LAB isolates derived from different sources indicated γ hemolysis (Hojjati et al. 2020; Mousanejadi et al. 2023; Zibaei‐Rad et al. 2023).

Infections can occur as a result of pathogens adhering to or infiltrating mucous membranes. However, probiotics can play a vital role in protecting the body against pathogenic infections (Behbahani and Noshad 2024). The production of antimicrobial compounds such as organic acids, diacetyl, hydrogen peroxide, carbon dioxide, and bacteriocin is an important property for probiotics to compete with pathogens (Behbahani et al. 2023). This antimicrobial effect, combined with the competitive exclusion mechanism, effectively prevents the colonization of pathogens in the intestinal tract (Echresh et al. 2024). All these results revealed that the degree of inhibition of LAB against test cultures was strain specific. Similar to our results, in a study performed by Akman et al. (2021), *L. fermentum* , *L. pentosus* , and *L. plantarum* derived from Shalgam juice exhibited inhibition zones ranging from 11.25 to 23.50 mm against *S. aureus* , *L. monocytogenes* , *E. coli* O157:H7, and *B. cereus*.

Starter cultures to be used in the production of probiotic foods with high salt content must be tolerant to high salt concentrations. In a study conducted by Samedi and Charles (2019), *E. faecalis* , *L. paraplantarum* , *L. plantarum* , and *W. paramesenteroides* isolated from leaves of food plants were viable at 2% salt, while they did not survive at 4% and 6.5% salt concentrations. Boricha et al. (2019) reported that *L. pentosus* , *L. pentosus* , and *L. fermentum* derived from fermented fruits survived at 4% and 6% salt concentrations.

The proteolytic enzyme activity of LAB is a significant technological characteristic that contributes to the improvement of the organoleptic features of fermented foods (Abubakr and Al‐Adiwish 2017). Lim et al. (2019) reported that *L. plantarum* strains isolated from tempeh and fermented cassava and *P. acidilactici* derived from tempeh exhibited proteolytic enzyme activity. In a study performed by Kivanc et al. (2011), the proteolytic enzyme activity of *E. faecium* , *L. plantarum* , *L. paraplantarum* , *L. brevis* , *Leuconostoc citreum* , *L. graminis* , and *L. lactis* isolated from fermented beverage (boza) was determined to be in the range of 0.04–2.50 mg tyrosine/mL.

Lactose intolerance is mainly due to a lack of β‐galactosidase, which hydrolyzes lactose into glucose and galactose. Therefore, food products fermented by probiotic strains with β‐galactosidase activity play a significant role in producing lactose‐free products in the treatment of lactose intolerance (Falah et al. 2021). Kumar et al. (2017) reported that *E. faecalis* , *Enterococcus* sp., *L. lactis* , *L. plantarum* , *L. mesenteroides* , and *P. pentosaceus* isolated from traditional pickles exhibited high levels of β‐galactosidase enzyme activities, and the highest enzyme activity was exhibited by *P. pentosaceus* PKL‐17, which was followed by *E. faecalis* PKL‐24. In another study, *L. delbrueckii* and *L. bulgaricus* strains derived from fermented beverages demonstrated high levels of β‐Galactosidase activity (Kumar et al. 2012).

The health‐promoting effects of probiotics depend on several mechanisms, including the ability of microorganisms to adhere to intestinal epithelial cells. The ability to adhere involves various interactions, including aggregation ability and cell hydrophobicity. Auto‐aggregation is an essential probiotic characteristic that demonstrates the ability of microbial cells to interact with each other (Rouhi et al. 2024). Furthermore, co‐aggregation of isolates indicates that a defensive barrier can be created to prevent the colonization of pathogens in intestinal cells (Echresh et al. 2024). Higher values were observed in a study carried out by Abouloifa et al. (2020), who reported that the auto‐aggregation ability of LAB strains ( *L. pentosus* , *L. plantarum* , and *L. brevis* ) ranged from 10.29% to 41.34%. Das et al. (2020) found that the auto‐aggregation ability of *L. plantarum* strains derived from fermented rice beverages ranged from 29.40% to 83.00%, while co‐aggregation percentages of strains with *S. typhi* were between 4.66% and 33.16% for 4 h. The co‐aggregation ability of LAB isolates with pathogens is an essential factor for maintaining healthy urogenital microflora. Besides, the ability of LAB strains for auto‐aggregation and co‐aggregation could facilitate the proliferation of probiotics in the GI tract (Das et al. 2020).

The adhesion of probiotics to the intestinal surface depends on their hydrophobicity. Strains' ability to adhere to epithelial cells can be determined by evaluating their adhesion to nonpolar solvents such as n‐hexadecane, xylene, and toluene (Falah et al. 2019). Our hydrophobicity values are higher than those reported in the literature. For example, Abouloifa et al. (2020) reported that the hydrophobicity of LAB strains derived from fermented green olives toward xylene ranged from 15.07% to 34.67%. In another study, the hydrophobicity values of *L. paracasei* strains isolated from fermented palm sap ranged from 24.16% to 68.18% toward xylene (Sornsenee et al. 2021). These results revealed that the cell surface hydrophobicity of isolates could vary considerably depending on the strain, isolation source, and hydrocarbons used.

Probiotics produce high short‐chain fatty acid (SCFA) production, including butyric acid and propionic acid, which adds unique functional value that could appeal to health‐conscious consumers. Pessione et al. (2015) specified that *L. plantarum* strains isolated from fermented olives produced 840–1180 μg/mL acetic acid, 0.61–2.89 μg/mL butyric acid, and 0.55–2.34 μg/mL propionic acid. In another study, the acetic, butyric, and propionic acid production of *W. paramesenteroides* FX5 and FX9 derived from fresh fruits ranged from 863 μg/mL to 6107 μg/mL, 16 μg/mL to 202 μg/mL, and 863 μg/mL to 3176 μg/mL, respectively (Pabari et al. 2020). It was observed that the production of acetic, butyric, and propionic acids by the strains isolated in the present study was considerably higher than these results. The results of these studies revealed that the strains produce various amounts of SCFAs depending on the strain.

## 5 Conclusions

Investigating new probiotic cultures has become significant because of the increasing utilization of probiotics in food and pharmaceutical products. Forty‐eight LAB strains were derived from commercial Shalgam juices and identified by 16S rRNA gene sequencing in the current study. The isolates belonged to eight LAB species, namely *E. faecalis* , *Lacticaseibacillus* sp., *Lentilactobacillus* sp., *L. brevis* , *L. buchneri* , *L. paracasei* , *L. parafarraginis* , and *P. acidilactici* . The isolates demonstrated high survival under GI tract conditions. The LAB strains also indicated different levels of susceptibility to antibiotics. The strains generally showed strong antimicrobial activity against the test microorganisms, and *E. coli* O157:H7 was the most delicate microorganism to LAB supernatants. Most of the strains indicated high hydrophobicity, auto‐aggregation, and co‐aggregation abilities, which demonstrate the adhesion of cells to the host tissue. The strains with the best probiotic features ( *P. acidilactici* SL7 and SL12, *L. paracasei* SL27) produced high levels of acetate, butyrate, and propionate. The results demonstrated that Shalgam juice could be a good probiotic source, as most of the LAB strains isolated from Shalgam juice had probiotic potential. Conversely, subsequent studies may encompass in vivo tests to substantiate the probiotic efficacy and health‐promoting attributes of the selected strains. However, the impact on gut microbiota diversity and metabolic activity should be investigated using animal models or human clinical trials. Finally, investigating the molecular mechanisms underlying the observed antimicrobial or functional properties may yield further information about their potential applications in the food and health industries. From an industrial standpoint, the strains isolated from Shalgam juice have the potential to be incorporated into functional foods, particularly vegetable‐based or non‐dairy probiotic formulations. Given the increased public interest in plant‐based and traditional fermented foods, these strains have promise for use in fermented vegetable products as starter cultures or as a functional food additive in free or encapsulated form. In this context, further research is required into their technological performance, stability under processing circumstances, and sensory impact in different foods. The isolates are already preserved in a culture collection with initial characterization completed, meaning that their further development, including in vivo health effects, can proceed more efficiently and at a lower cost. This positions them as economically advantageous candidates in comparison to commercially available probiotic strains, which often require licensing fees or de novo isolation and characterization processes. This cost‐effectiveness could enhance their appeal for industrial application, especially in local or regional markets focusing on traditional fermented foods.

## Author Contributions

**Ilkin Yucel Sengun:** conceptualization (equal), formal analysis (equal), investigation (equal), supervision (equal), validation (equal), writing – original draft (equal), writing – review and editing (equal). **Husniye Tansel Yalcin:** conceptualization (equal), formal analysis (equal), investigation (equal), validation (equal), writing – original draft (equal), writing – review and editing (equal). **Berna Ozturk:** conceptualization (equal), formal analysis (equal), investigation (equal), writing – original draft (equal), writing – review and editing (equal). **Aysegul Kirmizigul Peker:** conceptualization (equal), formal analysis (equal), investigation (equal), writing – original draft (equal), writing – review and editing (equal). **Gulden Kilic:** conceptualization (equal), formal analysis (equal), investigation (equal), writing – original draft (equal), writing – review and editing (equal). **Yigit Terzi:** conceptualization (equal), formal analysis (equal), investigation (equal), writing – original draft (equal), writing – review and editing (equal). **Yunus Yahsi:** conceptualization (equal), formal analysis (equal), investigation (equal), writing – original draft (equal), writing – review and editing (equal).

## Conflicts of Interest

The authors declare no conflicts of interest.

## Data Availability

Authors will make the availability of data and materials on reasonable request.
